# E-Tutoring

**DOI:** 10.1007/978-3-658-31279-4_20

**Published:** 2020-07-08

**Authors:** André Mersch, Alina Seibt

**Affiliations:** 1 Duale Hochschule Baden-Württemberg, Karlsruhe, Germany; 2 ILIAS open source e-Learning e.V., Köln, Germany; 3 Technische Hochschule Ostwestfalen-Lippe, Lemgo, Germany; 4 Duale Hochschule Baden-Württemberg, Karlsruhe, Germany; 5 Technische Hochschule Ostwestfalen-Lippe, Lemgo, Deutschland; 6 Technische Hochschule OWL, Lemgo, Deutschland

## Abstract

E-Learning bietet zahlreiche Möglichkeiten, die Präsenzlehre inhaltlich und didaktisch anzureichern und damit die Studienbedingungen zu verbessern. In der Praxis fehlt den Lehrenden jedoch oftmals der zeitliche Spielraum, um entsprechende Angebote zu erstellen. Das Konzept des E-Tutorings schafft hier Entlastung: E-Tutor* innen unterstützen Lehrende beim Einsatz digitaler Medien in der Gestaltung von Lehr-, Lern- und Prüfungsprozessen.

E-Learning bietet zahlreiche Möglichkeiten, die Präsenzlehre inhaltlich und didaktisch anzureichern und damit die Studienbedingungen zu verbessern. In der Praxis fehlt den Lehrenden jedoch oftmals der zeitliche Spielraum, um entsprechende Angebote zu erstellen. Das Konzept des E-Tutorings schafft hier Entlastung: E-Tutor*innen unterstützen Lehrende beim Einsatz digitaler Medien in der Gestaltung von Lehr-, Lern- und Prüfungsprozessen. Dabei helfen sie den Lehrenden, ihre jeweiligen Lehrveranstaltungen in den MINT-Fächern bedarfsgerecht auf einer Lernplattform einzubinden. Durch diese Entlastung kann der Zeitaufwand der Lehrenden für den Einsatz von E-Learning gering gehalten werden. Damit erhöht sich die persönliche Akzeptanz von E-Learning insgesamt und die Studierenden profitieren von den technischen und didaktischen Möglichkeiten der Lernplattform sowie vom wachsenden Angebot an Lern- und Übungsmaterialien für ihr Studium. In diesem Beitrag wird das Konzept des E-Tutorings in seiner Umsetzung an der TH OWL vorgestellt und durch Best Practice-Beispiele illustriert.

## 19.1 Das E-Tutoring an der TH OWL

In der gängigen Literatur wird der Begriff „E-Tutor“ (oder auch „Online-Tutor“) meist für Personen verwendet, die Online-Lernende während des Lernprozesses begleiten und betreuen (Rautenstrauch 2008, S. 10; Rakoczi und Herbst 2010, S. 131). Im optes-Projekt wird die Bezeichnung „E-Tutor“ bzw. „E-Tutorin“ für Personen verwendet, die in Abstimmung mit und im Auftrag von Dozent*innen, angeleitet durch eine*n als E-Tutoring-Koordinierungsstelle tätige wissenschaftliche*n Mitarbeiter*in, digitale Lehr- und Lernszenarien und -objekte entwickeln und bereitstellen.

Das E-Tutoring an der TH OWL ist das Ergebnis eines Prozesses, der bereits 2010 begann und im Zuge des optes-Projekts hochschulweit implementiert wurde. Am Beginn standen Tool-orientierte und mediendidaktische Workshops mit größeren Gruppen Lehrender. Die Evaluation dieser Maßnahmen ergab, dass eine Sensibilisierung Lehrender mit Blick auf die Potenziale des Einsatzes von Online-Medien in der Lehre stattfand, die Umsetzung jedoch häufig erst so versetzt zu den Workshops erfolgte, dass die vermittelten Umsetzungsschritte nicht mehr präsent waren. Vor diesem Hintergrund wurde ein auf die individuellen Bedarfe Lehrender abgestimmtes Beratungskonzept entwickelt (vgl. Lilienthal et al. 2020).

Jedem Unterstützungsprozess geht eine Abstimmung zwischen E-Tutoring-Koordinierung und Lehrenden voraus. Hier wird entlang von Veränderungsanlässen, die Lehrende in Ihren Veranstaltungen ausmachen, geprüft, wie diese umgesetzt werden können.

Dabei steht zunächst nicht der Einsatz von Online-Medien im alleinigen Fokus. Es wird unbedingt vermieden, E-Learning als Selbstzweck einzusetzen. Ergebnis einer ersten Beratung kann durchaus sein, dass bestimmte Anlässe nicht durch den Einsatz von Online-Medien, sondern durch analoge Methoden umgesetzt werden. Selbstverständlich liegt aufgrund des in das Digitale ausgerichteten Programms ein Fokus darauf, solche Verfahren zu verwenden, die mit Online-Medien und speziell der an der TH OWL vorhanden E-Learning-Infrastruktur umsetzbar sind. Es ist aber ein Anliegen, nicht nach dem Prinzip: „Wer einen Hammer in der Hand hält, sieht überall Nägel" vorzugehen, also Anforderungen stets mit den selbst präferierten, vorhandenen Lösungen zu begegnen, sondern die Perspektive offenzuhalten und dort, wo sie besser geeignet sind, analoge Methoden einzusetzen, bzw. diese eng mit einem digitalen Ansatz zu verknüpfen.

Dieses Prinzip folgt der Maßgabe, immer von Blended-Learning-Szenarien auszugehen. An einer Präsenzhochschule wie der TH OWL geht es nicht darum, reine Online-Verfahren einzusetzen, sondern Mehrwerte durch die Verknüpfung mit den Präsenzseminaren zu erzeugen.

Dabei orientiert sich der Beratungsprozess an dem Ebenen-Modell von Blended Learning nach Reinmann (Reinmann-Rothmeier 2003), das von einer Pyramide mit den Ebenen „Distribution", „Interaktion" und „Kooperation" ausgeht.

In den E-Tutoring-Beratungsprozessen ist es deshalb immer ein Ziel, nicht auf der reinen Distributionsebene zu verbleiben, auch wenn hier häufig der legitime Ansatzpunkt vieler Lehrender liegt, durch den Einsatz von Web-Werkzeugen Zeit im Lehrprozess zu sparen. Das ist der Ansatzpunkt, um von hier aus Mehrwerte mit und für die Lehrenden zu entwickeln, die sich durch ein Voranschreiten über die Pyramiden-Stufen ergeben.

Das Ebenen-Modell wird dabei flankiert durch das SAMR-Modell von Puentadura (2006), welches Lehrenden schrittweise Weiterentwicklungsmöglichkeiten aufzeigt. Dabei entstehen Szenarien der Modifikation von Lehrveranstaltungen bis hin zu neu definierten Lehrprozessen, wie dem Flipped-Classroom-Konzept folgende Umsetzungen.

Die E-Tutor*innen sind in diesem Prozess für die technische Umsetzung zuständig und bieten je nach Erfahrungsstand an dem zwischen der E-Tutoring-Koordinierung und den Lehrenden entwickeltem Konzept orientierte Weiterentwicklung an. Bei tiefer gehenden Bedarfen wird die E-Tutoring-Koordinierung erneut eingebunden, um die weitere Umsetzung festzulegen.

Lehrende werden insbesondere beim Umgang mit dem Lernmanagementsystem ILIAS durch E-Tutor*innen unterstützt. Die E-Tutor*innen zeigen den Lehrenden den Umgang mit der Lernplattform und wie diese sinnvoll in eine Lehrveranstaltung eingebunden werden kann. Das beinhaltet die Beratung zum Einsatz der vielfältigen Funktionen des Lernmanagementsystems ILIAS Darüber hinaus unterstützen sie sie im Umgang mit anderen Online-Werkzeugen, zum Beispiel zur Aufzeichnung von Lehrveranstaltungen, sowie Video- und Web-Konferenzen. Außerdem unterstützen sie bei der Abbildung und Organisation möglicher Kursangebote auf ILIAS und beraten bei allen Fragen rund um das Thema E-Learning. Nach der Einrichtung webbasierter Lerninfrastrukturen administrieren die E-Tutor*innen diese. Daher umfasst das typische Tätigkeitsspektrum zum Beispiel:

- Anleitung und Hilfe für Lehrende bei der Bedienung der Lernplattform als „Hilfe zur Selbsthilfe“
- Analyse und Vorbereitung der vorhandenen Lernmaterialien zum Bereitstellen auf der Lernplattform
- Unterstützung beim Einsatz von E-Learning-Werkzeugen, wie virtuellen Konferenzen oder Vorlesungsaufzeichnungen.

## 19.2 Die Ausbildung zum E-Tutor bzw. zur E-Tutorin

Die E-Tutor*innen werden in Form eines Kompaktseminars, wöchentlichen virtuellen Meetings und umfangreichen Online-Selbstlernaufgaben qualifiziert. Dabei werden didaktische Grundlagen des E-Learnings vermittelt, Anwendungsszenarien dargestellt, E-Learning-Werkzeuge behandelt und die technische Handhabung der Lernplattform eingeübt. Die Tätigkeit der E-Tutor*innen erfolgt im Rahmen einer Anstellung als studentische oder wissenschaftliche Hilfskraft. Alle Komponenten des Ausbildungskonzeptes sind im optes-Anwendungsnetzwerk herunterladbar.[1]

Um die für studentische Mitarbeitende herausfordernden Aufgaben zu bewältigen, werden E-Tutor*innen idealerweise in Tandems aus Erfahreneren und Einsteiger*innen eingesetzt. So wird nach der initialen Ausbildung neben den durch die E-Tutoring-Koordinierung angebotenen Weiterbildungsangeboten für die E-Tutor*innen eine implizite Weiterbildung durch Peer Learning gewährleitstet.

Die Studierenden sind auch deshalb in Tandems unterwegs, weil im Laufe des häufig mehrjährigen Einsatzes im E-Tutoring-Team eine Spezialisierung auf bestimmte Aufgabengebiete stattfindet. So gibt es zum Beispiel auf die Videobearbeitung spezialisierte E-Tutor*innen, andere haben sich auf bestimmte, sehr umfangreiche und tiefgreifende ILIAS-Funktionen, wie das Test- und Assess-

1 optes Anwendernetzwerk: http://anwender.optes.de [12.06.2020].

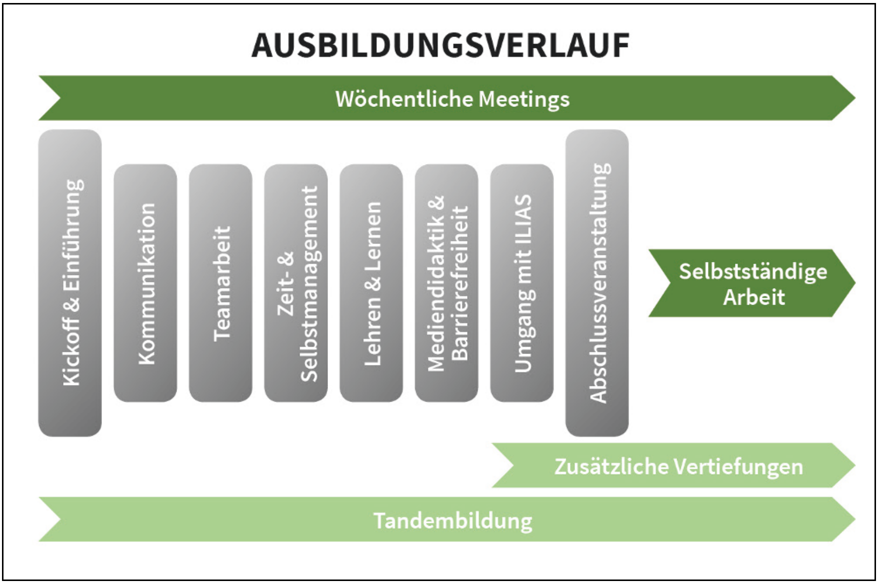

**Abbildung 1:** Ausbildungsprozess ment-Tool oder die enthaltenen Kommunikationsfunktionen, spezialisiert. Durch die Bildung von Tandems unterschiedlich spezialisierter E-Tutor*innen lässt sich eine sehr breite Begleitungsbasis schaffen.

Im regelmäßigen Austausch in E-Tutoring-Jour-Fixes, kann die E-Tutoring-Koordinierung so sehr effizient Beratungsverfahren unterstützen. Sie muss nicht bei allen E-Tutor*innen sicherstellen, dass diese jeweils über alle notwendigen Kompetenzen zur Unterstützung im jeweiligen Beratungsprozess verfügen, sondern kann die Teams so zusammenstellen, dass sie sich gegenseitig bei der Begleitung der Lehrenden unterstützen. So ist die gleichzeitige Begleitung von weit mehr Lehrenden möglich.

Auf diese Weise entsteht ein Pool von E-Tutor*innen, der kontinuierlich in Bezug auf Anzahl und Qualifikation der studentischen Mitarbeitenden auf einem Niveau gehalten wird und in dem sich die E-Tutor*innen im Peer Learning austauschen und weiterentwickeln können. Die Dokumentation aller Unterstützungsprozesse macht ein Wissens-Management möglich, bei dem alle Beteiligten voneinander lernen und zum Beispiel im Krankheitsfall die Prozesse von Kolleg*innen übernehmen können.

Das ist ein entscheidender Vorteil gegenüber einzelnen Einstellungen von studentischen Mitarbeitenden durch Lehrende, die in ihrer Aufgabe auf sich allein gestellt sind und mit der Beendigung des studentischen Mitarbeitendenverhältnisses auch ihr Wissen mitnehmen, das anschließend mit einem Nachfolger oder einer Nachfolgerin mühsam durch den Lehrenden wiederaufgebaut werden muss. Der berühmtberüchtigte „Brain Drain" wird durch das E-Tutoring-Konzept des Poolings vermieden.

## 19.3 Einsatz der E-Tutor*innen

Auch an der TH OWL liegt der Großteil der E-Learning-Szenarien im Bereich der Distribution, d. h. Lehrende setzen die Lernplattform vor allem ein, um ihre Materialien an die Studierenden zu übergeben und mit diesen zu kommunizieren. Diese Ebene dient dem E-Tutoring als Einstieg für didaktische Weiterentwicklungen gemeinsam mit den Lehrenden. Mit diesem Vorgehen ist es an der TH OWL gelungen, den Prozentsatz derjenigen Lehrenden kontinuierlich zu erhöhen, die nach einem ersten Mehrwerterleben auf der Distributionsebene von sich aus Szenarien ins Spiel bringen, die auf den Ebenen der Interaktion mit dem System und Kollaboration mit Hilfe des Systems liegen. Dabei ist es von entscheidender Bedeutung, den Lehrenden nicht nur eine konzeptionelle Beratung, sondern auch eine Umsetzungsunterstützung durch die E-Tutor*innen anbieten zu können. So wird vermieden, dass die vielversprechenden Konzepte am Zeitmangel des Arbeitsalltags scheitern.

Als Nebeneffekt gelingt es so, Lehrende auf der durch die Hochschule bereitgestellten Infrastruktur der Lernplattform und flankierenden Systemen, wie dem hochschuleigenen bzw. vom DFN-Verein bereitgestellten Web- und Videokonferenztools zu halten, die von Seiten des Datenschutzes und der Informationssicherheit überprüft sind. Dieses Tool-Angebot wird entlang der Bedarfe Lehrender kontinuierlich erweitert.

Durch die Einbettung in den E-Tutoring-Beratungsprozess kann leichter sichergestellt werden, dass Lehrende durch die gegebenenfalls unnötige Einbeziehung externer Systeme in die Falle laufen, dadurch Mehraufwände zu erzeugen, dass sie die notwendigen formalen Voraussetzungen zum Einsatz von Online-Systemen selbst herstellen, oder eines Tages feststellen müssen, dass das von ihnen genutzte Online-Werkzeug nicht mehr verfügbar ist.

Hier ist es von Vorteil, dass das E-Tutoring nicht nur in die Organisationseinheit zur didaktischen Beratung, sondern auch in die IT-Abteilung der TH OWL eingebunden ist. Dadurch ergibt sich ein in sich stimmiger, kontinuierlicher Verbesserungsprozess, der eine homogene Softwareinfrastruktur zur Folge hat. Von dieser profitieren Lehrende aufgrund der vorhandenen Pflege der Systeme und aktueller Dokumentation, sowie die Studierenden, die sich in einer Lernplattformumgebung mit einer einheitlichen Bedienlogik bewegen, sich also nicht von Tool zu Tool an neue Nutzungskonzepte gewöhnen und viele Benutzerkonten pflegen müssen.

*Best Practice „Distribution und Organisation“:*

In einem Lehrenden-Projekt wurde besonders das ILIAS-Lernmodul als Mittel zur Zeitersparnis und leichten Zugänglichmachung von Inhalten verwendet. In den Lehrveranstaltungen diente es aufgrund seiner Fähigkeit, multimediale Inhalte einzubinden, direkt als Präsentationsmittel, das live in der Veranstaltung bearbeitet und durch aktuelle Hinweise des Lehrenden sowie aus dem Austauschprozess in der Vorlesung ergänzt wurde. So entsteht gleichsam nebenbei eine Dokumentation des Lehrdialogs als gute Grundlage der Prüfungsvorbereitung.

*Best Practice „Interaktion“:*

Eine weitere Lehrenden-Beratung setzte auf die vorhandenen Materialien, wie Skripten und Präsentationen, auf und erweiterte diese um Selbsttest-Möglichkeiten für die Studierenden. So können Studierende nach den Vorlesungen anhand der auf der Lernplattform bereitgestellten Tests aus bis zu zwölf Fragetypen – von Single-Choice bis zu komplexen mathematischen Fragestellungen mit Zwischenlösungen (STACK-Frage: siehe Kapitel 10) – ihr Wissen überprüfen. Entsprechend des Blended-Learning-Prinzips bezieht der Lehrende die Ergebnisse der anonymen Tests in die Gestaltung der nachfolgenden Präsenzveranstaltungen ein. Wurden also zum Beispiel bestimmte Fragen von einer Vielzahl Studierender nicht korrekt beantwortet, wurde das Thema nochmals adressiert.

*Best Practice „Kollaboration“:*

Einige Lehrende haben in den letzten Jahren mit Hilfe der Begleitung durch das E-Tutoring komplexe E-Learning-Szenarien entwickelt, indem sie ihre Lehre entsprechend des Flipped-Classroom-Konzeptes (siehe Kapitel 20) gleichsam auf den Kopf gestellt haben. Dazu wurden Maßnahmen aus den Bereichen „Distribution“ und „Interaktion“ kombiniert. Hier kommen häufig Vorlesungsaufzeichnungen aus früheren Semestern zum Einsatz, die mit weiteren Objekten kombiniert werden. So stehen flankierend Selbsttests und Austauschforen zur Verfügung, gegebenenfalls ergänzt durch Abgabeverfahren für aufwändigere Ausarbeitungen, die nicht automatisiert, sondern durch Lehrende bzw. Tutor*innen redigiert werden. Die zugehörigen Präsenzveranstaltungen basieren auf der Bearbeitung der Materialien durch die Studierenden vor der Veranstaltung, sodass die Sitzungszeit für den informierten Austausch genutzt werden kann.

Besonders bei diesen Umsetzungen ist eine Begleitung auch der Studierenden bei der Nutzung der durch die Lehrenden aufgebauten Online-Infrastrukturen sinnvoll. Hier stehen an der TH OWL die Maßnahmen des optes-eigenen E-Mentoring sowie Betreuungskonzepte der Lernzentren zur Verfügung, die durch das Projekt „Praxis OWL" mit QPL-Mitteln aufgebaut wurden.

## 19.4 Marketing

Durch das durchweg hohe Interesse der Lehrenden an der TH OWL, die Potenziale des Einsatzes von Online-Medien in der Lehre kennenzulernen, war zu Beginn der Maßnahme kaum Marketing notwendig. Es galt das Prinzip der Unterstützung der Interessierten, entlang der S-Kurve, die die Kapazitäten des E-Tutoring ausschöpfen konnten. In der Projektlaufzeit wurde das Unterstützungsangebot des E-Tutoring durch ein kollegiales Austauschformat ergänzt, dass wiederum nicht ausschließlich die Möglichkeiten der Digitalen Lehre in den Blick nahm, sondern wiederum in Zusammenarbeit mit Projekt „Praxis OWL" – alle didaktischen Facetten der Hochschullehre adressierte. Unter dem Namen „Pickert, Prütt und Plausch" werden im Wechsel Verfahren der digitalen Lehre und analoge Methoden adressiert, indem jeweils externe Inputgeber*innen berichten und durch Lehrende der Hochschule ergänzt werden. Im Rahmen dieser gestalteten Mittagspause liegt der Fokus auf dem an die Inputs anschließenden Austausch der Lehrenden untereinander und mit den externen Inputgeber*innen.

Auf Wunsch von Lehrenden wurde diesem Format die sogenannte „eCampus Coffee Break" zur Seite gestellt, deren Fokus ganz auf der Betrachtung der Möglichkeiten der ILIAS-basierten TH-OWL-Lernplattform eCampus ausgerichtet ist. Hier werden Good Practices ausgetauscht und eine stets wachsende Community of Practice der digitalen Lehre gepflegt.

Erweiterte Marketing-Maßnahmen des E-Tutoring sind die besondere Berücksichtigung von Leistungen im Bereich der digitalen Erweiterung der Lehre bei der jährlichen Vergabe des hochschulweiten Lehrpreises, die Möglichkeit zur Beteiligung an Publikationen, zum Beispiel im Rahmen der hauseigenen Herausgeberreihe „Teaching Exchange", sowie die Begleitung Lehrender bei der Erstellung von Förderanträgen im Bereich der digitalen Lehre.

## 19.5 Fazit – Übertragbarkeit an andere Hochschulen

Die Pilotierung und Weiterentwicklung des optes-E-Tutoring an der TH OWL liefert ein gelungenes Beispiel für die schrittweise Digitalisierung der Lehre. Durch den Brückenschlag zwischen den optes-Vorkursen und der Begleitung Lehrender bei der Gestaltung ihrer grundständigen Lehre mit digitalen Medien ist eine ganzheitliche, zukunftsfähige Lehrstrategie mitentwickelt und umgesetzt worden. Besonders das Zusammenspiel aus konzeptioneller Beratung und Umsetzungs-unterstützung ist dabei ein wesentlicher Erfolgsfaktor. Durch die gelebte „Hilfe zur Selbsthilfe“ ist ein robuster Sockel Lehrender entstanden, die ihre Lehre mit digitalen Elementen anreichern und diese auch weitergehend didaktisch reflektieren und modifizieren. Durch die Community of Practice sogenannter „Super User“ in den Fachbereichen breitet sich der Einsatz digitaler Medien unter den Lehrenden stetig weiter aus.

Die im Rahmen des Projekts entwickelten frei verfügbaren Komponenten des E-Tutoring sind vor allem deshalb für andere Hochschulen interessant, weil sie sich wie das optes-Mathematik-Material an die spezifischen Bedarfe und Rahmenbedingungen anderer Hochschulen anpassen lassen.

Unter dem Eindruck der Präventionsmaßnahmen im Rahmen von Covid-19 wurde besonders deutlich, wie stark die durch das E-Tutoring mitgeschaffene technische und beraterische Infrastruktur trägt. Binnen kürzester Zeit konnten die für den Einsatz mit besonders interessierten Lehrenden entwickelten Materialien und Verfahren so um- und ausgebaut werden, dass eine Vielzahl von Lehrenden schnell dabei unterstützt werden konnte, im digitalen Raum handlungsfähig zu werden.

## Weiterführende Literatur

Knaus, T., Meister, D. & Tulodziecki, G. (2018). Qualitätsentwicklung – Professionalisierung – Standards. Thesen aus medienpädagogischer Sicht. In T. Knaus, D. Meister & G. Tulodziecki (Hrsg.), *Futurelab Medienpädagogik. Qualitätsentwicklung, Professionalisierung, Standards* (S. 23-47). München: kopaed.

Knaus, T., Meister, D. & Narr, K. (2018). Digitalisierung erfordert professionelle medienpädagogische Unterstützung. In T. Knaus, D. Meister & G. Tulodziecki (Hrsg.), *Futurelab Medienpädagogik. Qualitätsentwicklung, Professionalisierung, Standards* (S. 211-213). München: kopaed.

Schäffer, D. & Osterhagen, T. (2016). *Lernmanagement-Systeme mit Konzept einsetzen – Lehrende und Studierende beim Online-Lernen begleiten*. Tübingen: e-teaching.org.

Schäffer, D. (2019). Studierende begleiten Professor*innen – Ausbildungskonzept für studentische E-Learning-Berater*innen. In T. Schmohl & K. To (Hrsg.), *Hochschullehre als reflektierte Praxis. Fachdidaktische Fallbeispiele mit Transferpotenzial, 2. Aufl.* (S. 57–68). Bielefeld: wbv.

**Open Access** Dieses Kapitelwirdunter der Creative Commons Namensnennung 4.0 International Lizenz (http://creativecommons.org/licenses/by/4.0/deed.de) veröffentlicht, welche die Nutzung, Vervielfältigung, Bearbeitung, Verbreitung und Wiedergabe in jeglichem Medium und Format erlaubt, sofernSie den/die ursprünglichenAutor(en) und die Quelleordnungsgemäßnennen, einen Link zur Creative Commons Lizenzbeifügen und angeben, obÄnderungenvorgenommenwurden.

Die in diesemKapitelenthaltenenBilder und sonstigesDrittmaterialunterliegenebenfalls der genannten Creative Commons Lizenz, sofernsichaus der Abbildungslegendenichtsanderesergibt. Sofern das betreffende Material nichtunter der genannten Creative Commons Lizenzsteht und die betreffendeHandlungnichtnachgesetzlichenVorschriftenerlaubtist, istfür die obenaufgeführten Weiterverwendungen des Materials die Einwilligung des jeweiligenRechteinhaberseinzuholen.
